# Suppression head impulse test in children—experiences in a tertiary paediatric vestibular centre

**DOI:** 10.3389/fneur.2024.1297707

**Published:** 2024-03-14

**Authors:** Soumit Dasgupta, Rosa Crunkhorn, John Wong, Annie McMahon, Sudhira Ratnayake, Leonardo Manzari

**Affiliations:** ^1^Department of Paediatric Audiology and Audiovestibular Medicine, Alder Hey Children’s NHS Foundation Trust, Liverpool, United Kingdom; ^2^Faculty of Health and Life Sciences, University of Liverpool, Liverpool, United Kingdom; ^3^Department of Otorhinolaryngology, Guys Hospital, London, United Kingdom; ^4^MSA ENT Academy Centre, Cassino, Italy

**Keywords:** video head impulse test, suppression head impulse test, pediatrics, vestibulo-ocular reflex gain, peak saccadic velocity, vestibular compensation, children, latency

## Abstract

The suppression head impulse paradigm (SHIMP) involves suppression of the vestibulo-ocular reflex (VOR) and anticompensatory saccades generated thereof. SHIMP is gaining importance to understand vestibular compensation with its different parameters (VOR gain/peak saccadic velocity PSV/latency of saccades). SHIMP studies are emerging in adults, but pediatric studies have hardly been performed. This study is a retrospective case note audit over a period of 2 months in a tertiary pediatric vestibular center in the United Kingdom to investigate whether SHIMP is safe/robust to be used in children conforming to existing standards/norms in normal children and whether it yields any meaningful inferences in pediatric vestibular hypofunction. This is the largest pediatric SHIMP study to date. A total of 44 referred children (6–18 years, female children>male children) with a range of complaints from dizziness, imbalance, motor incoordination, postural instability, and hearing loss were included, and their SHIMP parameters were measured. All children underwent comprehensive functional/objective audiovestibular assessments. Two groups were defined—Group A with normal vestibular function and Group B with abnormal vestibular function. The normal population showed an average SHIMP VOR gain of 0.98+/−0.08 and latency of overt saccades at 215.68+/–46.16 milliseconds agreeing with published evidence. The PSV of overt saccades was 315.39+/−56.3^0^/s, and there was a gain asymmetry of 7.42+/−4.68 between the sides. Statistically significant differences with moderate/large effect sizes were observed between the groups in terms of VOR gain and PSV but not in saccade latencies. Covert saccades were rare in SHIMP, while overt saccades were observed in 100% of children. VOR gain difference between the head impulse paradigm (HIMP) and the SHIMP was significant as well. We observed statistically significant differences in side asymmetry of VOR gain between the groups. Furthermore, we identified a group of children with cerebellar lesions where overt saccades in SHIMP were rather low in number. Further research is recommended to investigate pediatric PSV, asymmetry, and inability to generate overt saccades that may suggest useful means to assess compensation and central function. We conclude that SHIMP yields valuable information and is a safe, easy to perform, and a reliable test that should be used in children to supplement HIMP.

## Introduction

The head impulse test first enumerated by Halmagyi and Curthoys (1) ushered in a new era of vestibular diagnostics. It paved the way for quantifying high frequency semicircular canal function in all six canals, the high frequency that we primarily use when we move our heads. Therefore, the practical value of this test to assess semicircular canal function cannot be emphasized enough. In the normal situation, in response to a rapid acceleration head thrust on one side, the eyes of the subject remain focussed on a fixed target by moving to the opposite direction to the head movement, a movement generated by the vestibulo-ocular reflex (VOR). In vestibular weakness, the eyes are unable to match the head movement and the central compensatory mechanism generates a quick catch-up movement called a compensatory saccade to the opposite side of the head movement that can be seen by the examiner and indicates angular canal dysfunction.

The first attempt to physically measure the response to the head impulse was by Ulmer and Chay where they used a remote mounted camera (2). Measurement was refined by the MacDougal team in 2009 when they measured the head impulse response with a head mounted camera and compared the video-oculography with a gold-standard scleral coil to show robust concordance (3). This technique was called the video head impulse test or vHIT and the protocol used for the test called the Head Impulse Protocol or HIMP. With this technique, the VOR gain (defined as the ratio of the velocity of the eye movement to the head movement), the peak saccadic velocity (PSV), the latency, and the occurrence of saccades relative to the head thrusts (the number of times that saccades are present in a given number of head thrusts) could be objectively measured. An additional parameter that of a covert saccade was discovered that cannot be seen by the clinical head impulse test as this saccade is generated during the head movement. This covert saccade also indicates a canal dysfunction. Intuitively, it can be inferred that the amplitude of the compensatory overt saccade (i.e., the saccadic velocity) is proportional to the degree of weakness of the VOR, i.e., more the weakness, more is the amplitude of the saccade (4). Furthermore, it has emerged that in compensating vestibular weakness, the VOR gain might recover without saccades (5), but saccades may also persist (6) due to prolonged central adjustment regulating mechanisms.

Since its discovery, the video head impulse test with the HIMP protocol has been adopted as a key vestibular high frequency canal diagnostic function to diagnose peripheral angular motion deficits and has been subjected to intense research. The sensitivity and the specificity of the test vary from 88 to 100% (3, 7). Finer aspects of the HIMP protocol beyond measuring the adult peripheral vestibular system are emerging, for example the HIMP in central neurological lesions (8). In the pediatric population, the video head impulse test with the HIMP protocol is gradually emerging but even then, it is limited (9). It is a safe and user-friendly procedure to perform in children with good information about canal function but requires a high skill set.

While the VOR is an essential reflex to prevent retinal slip during natural daily head motions, it is opposite to the eye movements in naturally occurring head movements where the subject follows a moving target and is thus counterproductive in these situations. A mechanism called VOR suppression or cancellation therefore exists that is a voluntary effort to redirect the gaze in the same direction as the moving target that overrides the VOR. The suppression is regulated by central mechanisms and is subjected to individual variations. Suppression shows visually dependent (driven by the smooth pursuit system) and visually independent (driven by non-visual higher center processes) characteristics (10). The suppression rarely occurs before the onset of visual pursuit and on an average is between 80 and 120 milliseconds after the head movement is initiated (11).

In 2016, the MacDougal team proposed a new test based on the video head impulse test, the suppression head impulse test (SHIMP) (12). In this test, the subject does not fixate gaze on a stationery or earth fixed target but rather directs gaze to a moving target that is head mounted. In the normal situation, the VOR that is set up in response to a head movement on one side directed to the opposite side of the movement will be suppressed or cancelled when the eyes follow the moving target. At the end of the head movement, to maintain foveation on the target, a large saccade will be generated that being directed to the same side of the movement is dubbed as an anticompensatory saccade. In the abnormal situation with vestibular weakness, the VOR will be weak or absent in which case, the eyes will naturally follow the moving target in the same direction and thus the production of an anticompensatory saccade is not or less obviated. This leads to the observation that with total VOR deficit, there will be no anticompensatory saccades. Such saccades will start to reappear with recovery of VOR and therefore will indicate compensation. As like the HIMP, VOR gain, saccade occurrence, PSV, and relative asymmetry between the two sides can be objectively measured.

In a recent review of literature (13) on SHIMP, it was observed that the test not only complemented the HIMP but also yielded better values on the VOR gain as contaminating covert saccades contributing to VOR gain are far less. PSV and the percentage of saccades indicate compensating vestibular function which are useful to glean an idea about central pathways that govern the suppression mechanism. The sensitivity and specificity are postulated to be nearly 100% to diagnose a vestibular weakness (13). SHIMP behaves differently in uncompensated and compensated vestibular deficits and may be useful to plan a rehabilitation program.

We emphasize that although a vestibular weakness in a child undergoes robust compensation and may render the child relatively asymptomatic, diagnosis is still essential with objective testing. In this respect, the SHIMP test that indicates vestibular compensation in various stages more than anything else might have a very important role to play. This is essential for a holistic management approach to manage disorders of balance function in children where cognitive and situational counseling are pivotal for both children affected and their carers/parents to maximize favorable outcome. Managing children with such disorders is quite different from what is practiced in adults due to the important premise that the vestibular system takes a significant part in overall development of children in several domains including motor skills, playground activities, navigation, and cognition (14).

The SHIMP paradigm has hardly been studied in children. The physiology/pathology and behavior of pediatric and adult vestibular function in health and disease differ from each other (9). Cerebral plasticity in children ensures robust compensation, and thus, it is important to figure out how SHIMP will behave in children. The present study is the largest study to date assessing SHIMP in children comprising of both a normal and a pathological cohort of children.

## Patients and methods

### Patients

A retrospective case note audit over a period of 2 months including a reaudit was performed in children who were referred to the tertiary audiovestibular department in Alder Hey Hospitals in Liverpool, UK, with complaints of dizziness/vertigo, imbalance/incoordination and sensorineural hearing losses. The first audit included children assessed in October 2019, and the reaudit was undertaken including children assessed in January 2023. There was no set exclusion or inclusion criteria except children with concomitant oculomotor pathologies were excluded as such disorders can contaminate the video head impulse test. All children referred were included as we wanted to study SHIMP feasibility and parameters on all children regardless of a vestibular weakness or not to incorporate the test in routine clinical practice. The audit was registered at and approved by the clinical audit department of the Alder Hey Children’s NHS Foundation Trust, hospital registration number 5941 and 6960. Being an audit, the study was exempted from formal Research Ethics Committee approval as stipulated in the UK.

An audit is essentially a service improvement exercise, comparing current practices with established practices and observing the compatibility and concordance between the two. Our audit standards were formulated on the data given in the two publications on SHIMP (15, 16) in children in the normal population and on numerous publications in the adult population that have established the utility of SHIMP in the normal and in the abnormal population. We wanted to explore whether normative data in children agree with our observations. In addition, we inferred that in children with vestibular deficits, the parameters observed in SHIMP will differ significantly from the normal population as observed in adults which we listed in our audit standards as well.

The aim of this study is to quantify SHIMP parameters in children with normal and abnormal vestibular function in terms of VOR gain, PSV, incidence of saccades, ease of use in children, and asymmetry between the two sides. The objectives were to define normative values of SHIMP parameters for subsequent audit benchmarks and analyse the agreement with previously published audit standards. A further objective was whether the SHIMP paradigm is fit for purpose and is a useful supplement to the standard HIMP paradigm in children as a part of quality assurance of vestibular diagnostic methods.

Audit standards are given in Table 1.

**Table 1:** Audit standards.

Normal population	Population with abnormal vestibular function
SHIMP gain between 0.94 (Left) and 0.99 (Right) SHIMP VOR gain statistically less than HIMP VOR gain Saccades present in at least 90% of subjects Latency of saccades from 166 milliseconds (Left) to 129 milliseconds (Right) PSV normal Range of asymmetry of VOR gain between normal and abnormal side normal Easy to use	SHIMP VOR gain statistically less than normal VOR gain SHIMP VOR gain significantly different from HIMP VOR gain Saccades present in at least 90% Latency of saccades as normal group PSV abnormal Range of asymmetry of VOR gain between normal and abnormal side abnormal Indicative of vestibular compensation Easy to use

## Methods

### Anamnesis

All children underwent a full anamnesis that included history of vestibular behavior from the carers/parents. In addition, behavior in terms of balance, coordination, movement, playground activities, and hearing losses were enquired upon. Full developmental, physical, past history of head injuries, medication history, motor skills, school performance, behavior, and social interaction issues were documented. Audiovestibular history in children is required to be holistic. The points in history are given in Table 2.

**Table 2:** History for peripheral and central pediatric vestibular deficits.

Obvious disorientation in space described by older children
Bumping into objects/door frames/navigational difficulties Clumsiness and incoordination Sudden vacant looks with very brief lasting falls followed by immediate complete recovery Migrainous features and/or vomiting Delayed motor development and abnormal motor skills (running, swimming, dancing, etc.) Walking difficulties, unsteadiness, postural instability, ataxia Sensation problems and dysarthria Difficult balance in darkness, slippery, and uneven surface World jumping in front of eyes Difficulties in riding a bike Difficulties in playground activities and amusement park rides Vestibular behavior observed up by others (care giver/school) Difficulties in challenging visual environments Poor reading skills and/or head eye or hand eye coordination Hearing loss, aural symptoms Third window symptoms if described by older children (conductive dysacusis, gaze evoked tinnitus, Tullio and Hennebert’s phenomenon, autophony) General symptoms – tiredness, cognitive/psychological symptoms, behavioral issues, syncopal spells School performance and social interaction Birth, family, trauma, ototoxicity, and development history

### Audiological assessment

All children underwent otoscopy, tympanometry, acoustic reflex tests, transient otoacoustic emissions, and pure tone audiometry following the British Society of Audiology standards. Aurical Audiometer, Otometrics Zodiac, and Otodynamics equipment were used.

### Vestibular assessment

All children underwent a full set of videonystagmography (VNG) examination with and without optic fixation incorporating observation of nystagmus, smooth pursuits and saccades in the horizontal and the vertical directions, head shake, head heave, ocular counter roll, the mastoid vibration test, the office rotatory chair test, the VOR suppression test; the full set of vestibulospinal test battery with and without proprioception and visual fixation including the Romberg, the sharpened Romberg, the Unterberger and the tandem gait tests, and clinical assessment of the subjective visual vertical.

Next, they underwent the video head impulse test with both the HIMP and the SHIMP paradigms. For the HIMP paradigm, HIMP for all six canals was performed. For the SHIMP paradigm, only the lateral semicircular canals were performed as current technology permits SHIMP only for lateral semicircular canals. The current study investigated the SHIMPs. For the HIMP and the SHIMP paradigms, 5–10 random and unpredictable head thrusts were executed on each side by the first author who is an experienced pediatric vestibular specialist and who has performed over 1,000 vHITs in children to maintain consistency. Unpredictability was achieved by modified distraction techniques between the head impulses with parent participation whenever required. The equipment used was ICS Impulse 2019 version. For SHIMP, incidence of saccades, PSV, VOR gain, saccade latency, and asymmetry between the two sides were recorded. We did not consider saccade clustering and PR score as these parameters are still being researched and experimental without much consensus especially in children. We had already established our HIMP norms where VOR gains range from 0.9 to 1 in horizontal canals and 0.6 to 0.8 in vertical canals between the ages 6 and 18 years (14). All the HIMP and SHIMP outputs were analyzed by two senior pediatric audiovestibular physicians, and agreement was reached in terms of validity, exclusion of artifacts, and presence of meaningful saccades.

Finally, the children underwent a cervical-evoked vestibular myogenic potential test (cVEMP) with Neurosoft 2019. Our VEMP laboratory norms include asymmetry up to 26% and thresholds of 80 ≥ dBnHL between the ages of 4 and 16 years (14).

We did not perform caloric testing due to the distress it causes in children. In our center, we additionally used the mastoid vibration test that is a child friendly test and that shows a good correlation with the caloric test (17) although it tests a different frequency response of the vestibular sensory epithelium as compared to the calorics. Dix Hallpike, supine head roll, and deep head hanging tests were undertaken when indicated.

### Other assessment

Full developmental, physical, oculomotor, musculoskeletal, cardiological, and neurological examinations of all children were performed.

The full battery is given in Table 3.

**Table 3:** Pediatric audiovestibular test battery.

Audiological tests	Vestibular tests
Pure tone audiometry with masking Tympanometry Stapedial reflexes Otoscopy Transient otoacoustic emissions	Full developmental examination Full neurological examination Musculoskeletal examination Cardiological examination Full oculomotor examination Assessment of subjective visual vertical VNG with and without visual fixation for smooth pursuits/saccades, head shake, head heave, ocular counter rolling, mastoid vibration, and ectopic eye movements Video head impulse test Suppression Head Impulse test Cervical vestibular evoked myogenic potential test Vestibulo-spinal test battery with and without proprioception for Romberg, Unterberger, tandem gait; one legged stance and sharpened Romberg Office rotatory chair tests and suppression of visual fixation BPPV tests

### Statistical methods

Depending on vestibular function test results, two groups were defined: Group A: Children with normal vestibular function test battery were deemed normal in terms of vestibular involvement and Group B: Children with physically quantified abnormal peripheral or central vestibular function were deemed abnormal in terms of vestibular involvement. Additionally, two subgroups of Group B were defined: Group B1 where HIMP VOR gain was normal but catch-up saccades were present and Group B2 where overt saccades in SHIMP were grossly reduced in number.

Descriptive and comparative statistics were computed by Social Statistics 2023.^1^ Comparison between the groups involved the *t*-test with a confidence interval of 95% along with calculation of effect sizes. A *p*-value of < 0.05 was deemed as statistically significant. Effect sizes *d* up to 0.2 were considered small, up to 0.5 were considered moderate, and up to 0.8 or more were considered large.

## Results

### Execution of procedure

All children in the cohort of 44 subjects across all age groups performed the SHIMP test with meaningful results. The average time taken was *circa* 2 min. Most children found it engaging and easier to follow a moving target rather than a fixed target as in HIMP. Special considerations for children were listed as apprehension, difficult fixation and unstable gaze, long eyelashes, and position of hands on the head. With practice and employing a fun and play technique, these problems can be mitigated with correct placement of the goggles. The first author observed that performing the SHIMP test takes more practice than the HIMP test as the children must be instructed properly how to perform the test.

### Demographics

**Figure 1:**
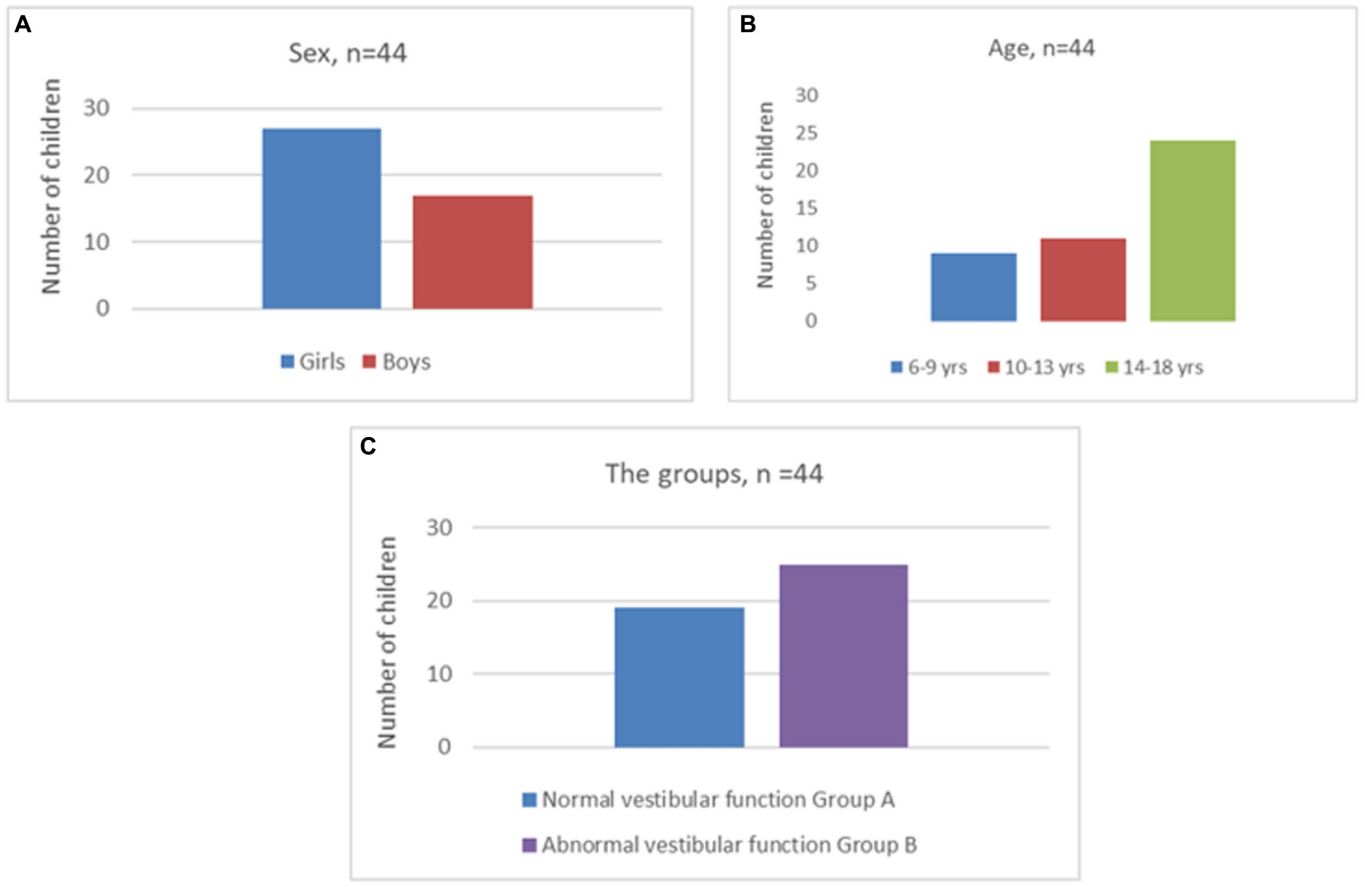
Sex **(A)**, age distribution **(B)** and distribution of groups **(C)**.

There were 44 children in the study with 17 boys and 27 girls with an average age of 12.7 years over a range of 6–18 years. The distribution is given in Figures 1A,B.

### Etiology

Presenting complaints included reliable history of vertigo/dizziness, imbalance and motor coordination deficits, ataxia, and sensorineural hearing loss. The relative etiology is given in Table 4. Figure 1C defines the normal and the abnormal group. In total, 43% of the cohort did not return any abnormalities in the comprehensive peripheral and central vestibular test battery (the normal Group A), while 57% showed some abnormalities (the abnormal Group B). The normal group included vestibular migraine, motor skill deficits/development coordination disorders, lower limb biomechanical abnormalities, and 27% of sensorineural hearing losses (4/11). Vestibular migraine was the commonest etiology in children presenting with dizziness constituting 25% of the entire cohort.

**Table 4:** Etiology.

Etiology	Frequency
Lower limb biomechanical abnormalities – pes planus, hyperelasticity, leg length discrepancies	2
Tumor/Space occupying lesions in cerebellopontine angle and intratympanic regions - arteriovenous malformations in posterior fossa cerebellopontine angle, facial nerve neuroma, cross arterial compression in cerebellopontine angle	4
Structural vestibular lesions – vestibular nerve hypoplasia	1
Central lesions affecting vestibular nerve bundle - Arnold Chiari malformation, intracranial ependymoma	2
Vestibular migraine	11
Sensorineural hearing loss – idiopathic, genetic, ototoxicity, head injury, posterior fossa tumors	15
Central lesions with development coordination disorder/ataxic syndromes – CACNA1 mutation, cerebral palsies, Stickler syndrome, and idiopathic	7
Congenital cochleovestibular syndromes – X linked gusher, FLNB mutation	2
Postural dizziness	1
Vestibular neuritis	2
Head injury with cochleovestibular involvement	1

### Head velocity of SHIMP head thrusts, *n* = 88 ears in 44 subjects

**Figure 2:**
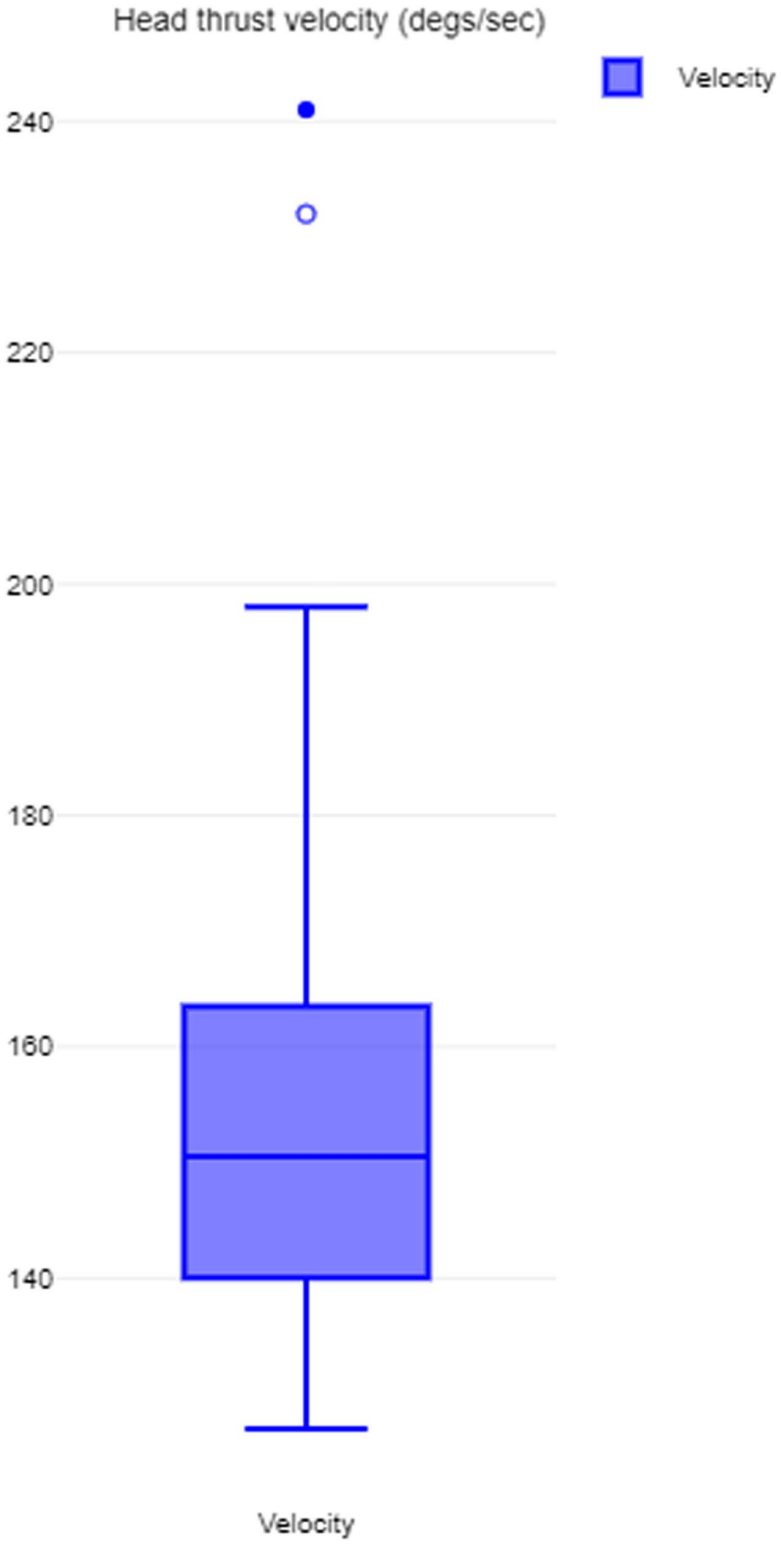
SHIMP head thrust velocity.

The average head velocity of the head thrusts for SHIMP was 154.34 degrees/s +/− 20.68. This is graphically represented in Figure 2. As can be seen that the velocities were contained within a compact range without significant outliers as they were consistently performed by one examiner.

### VOR gain in SHIMP

**Figure 3:**
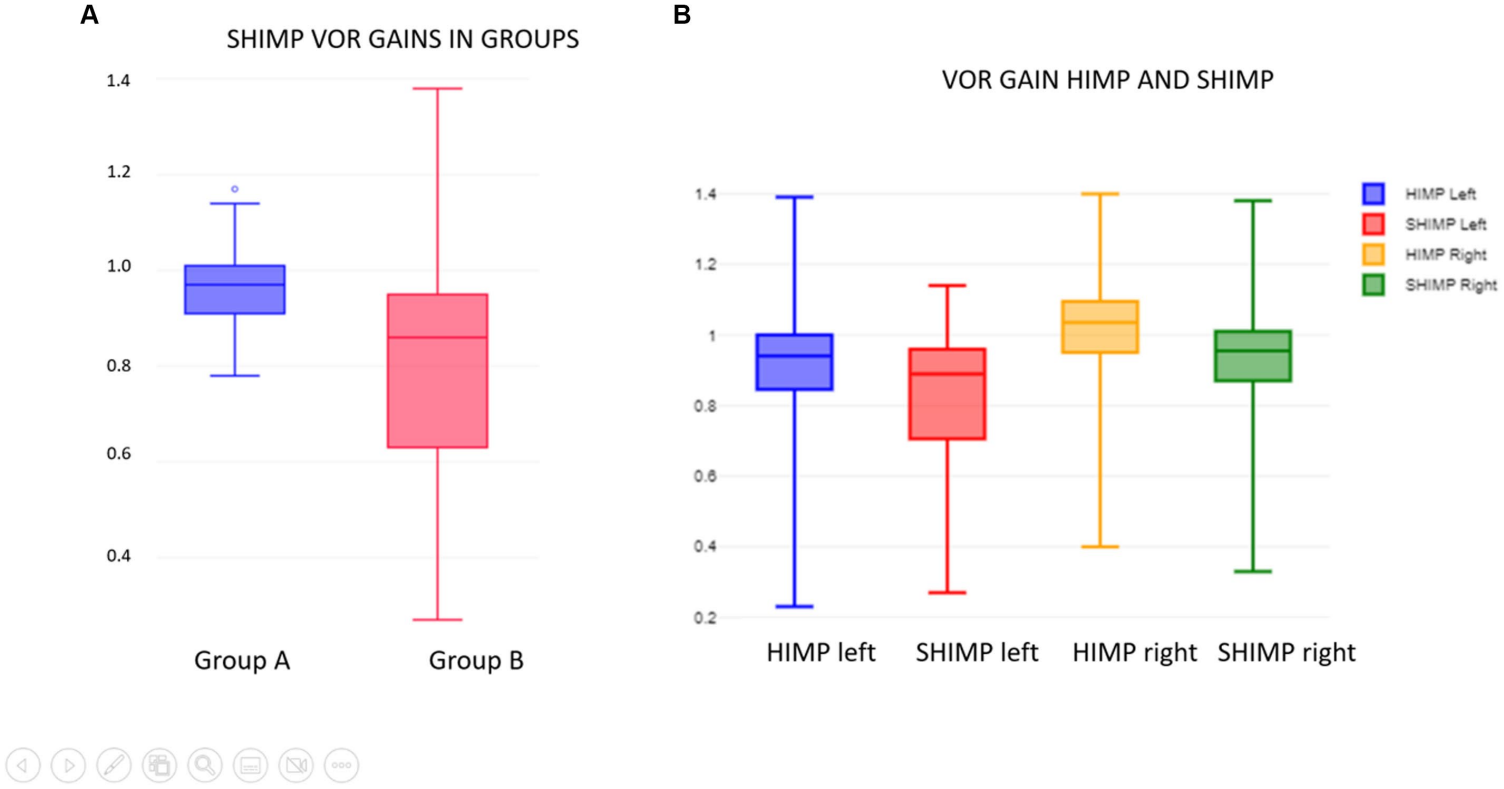
**(A)** SHIMP VOR gain in groups A and B. **(B)** VOR gain in HIMP and SHIMP.

The average VOR gain in SHIMP on the left in Group A was 0.95 +/− 0.08 and on the right was 1 +/− 0.08. Both sides combined, and the average gain was 0.98 +/− 0.08 In Group B, on the left it was 0.76 +/−0.18 and on the right it was 0.87 +/−0.25. Both sides combined, and the VOR gain was 0.81 +/−0.22. When the groups were compared, the difference was statistically significant with *p* = 0.000019 and *d* = 1 indicating a large effect size. Furthermore, it can be seen that the VOR gain in Group B is spread over a wider range. Figure 3A and Table 5 show this.

**Table 5:** SHIMP measured parameters (significant differences with *p* < 0.05 in italics) *n* = 44.

	VOR gain L	VOR gain R	R + L VOR gain	Peak saccadic velocity (degs/s) overt saccades	Overt percentage	Overt latency in milliseconds	Asymmetry	Covert percentage and latency
Group A	0.95+/−0.08	1+/−0.09	*0.98+/−0.08*	*315.39+/−56.3*	93.87+/8.730	215.68+/46.16	*7.42+/−4.68*	6.89+/−11.21; 113.29+/−24.66 ms
Group B	0.76+/−0.18	0.87+/−0.25	*0.81+/−0.22*	*293.6+/−56.76*	92.32+/10.64	216.74+/37.54	*21.12+/−14.42*	10.52+/−15.57; 111.63+/−21.26 ms

The average VOR gain in HIMP on the left combining both groups was 0.9 +/− 0.18 and compared to the SHIMP VOR gain on the left combining both groups was statistically higher and very close to significance with *p* = 0.52 with a moderate effect size. On the right considering similar measurements, the HIMP VOR gain was 1.01 +/− 0.17 and compared to the SHIMP VOR gain on the left was statistically higher and significant at *p* = 0.02 with similar effect size. Figure 3B and Table 6 summarize the VOR gain observations in SHIMP and HIMP.

**Table 6:** Comparison of HIMP and SHIMP VOR gains, *n* = 44.

	VOR gain left	VOR gain right	95% CI and effect size
HIMP	0.9+/−0.18	1.01+/−0.17	*p* = 0.052 left*d* = 0.3 left *p* = 0.02 right *d* = 0.4 right
SHIMP	0.84+/−0.17	0.92+/−0.2	

### Generation of covert and overt saccades

Covert saccades in SHIMP were generated in 28 out of the 88 ears tested, i.e., in 31% of the ears tested, but the percentage of covert saccades in each subject was rather low with the children generating such saccades on an average 8.7% times during the head thrusts. Importantly, in children with low VOR gain and overt/covert saccades in HIMP indicating peripheral vestibular weakness (n = 7), SHIMP covert saccades were not generated in 70% on the sides of the lesion.

**Figure 4:**
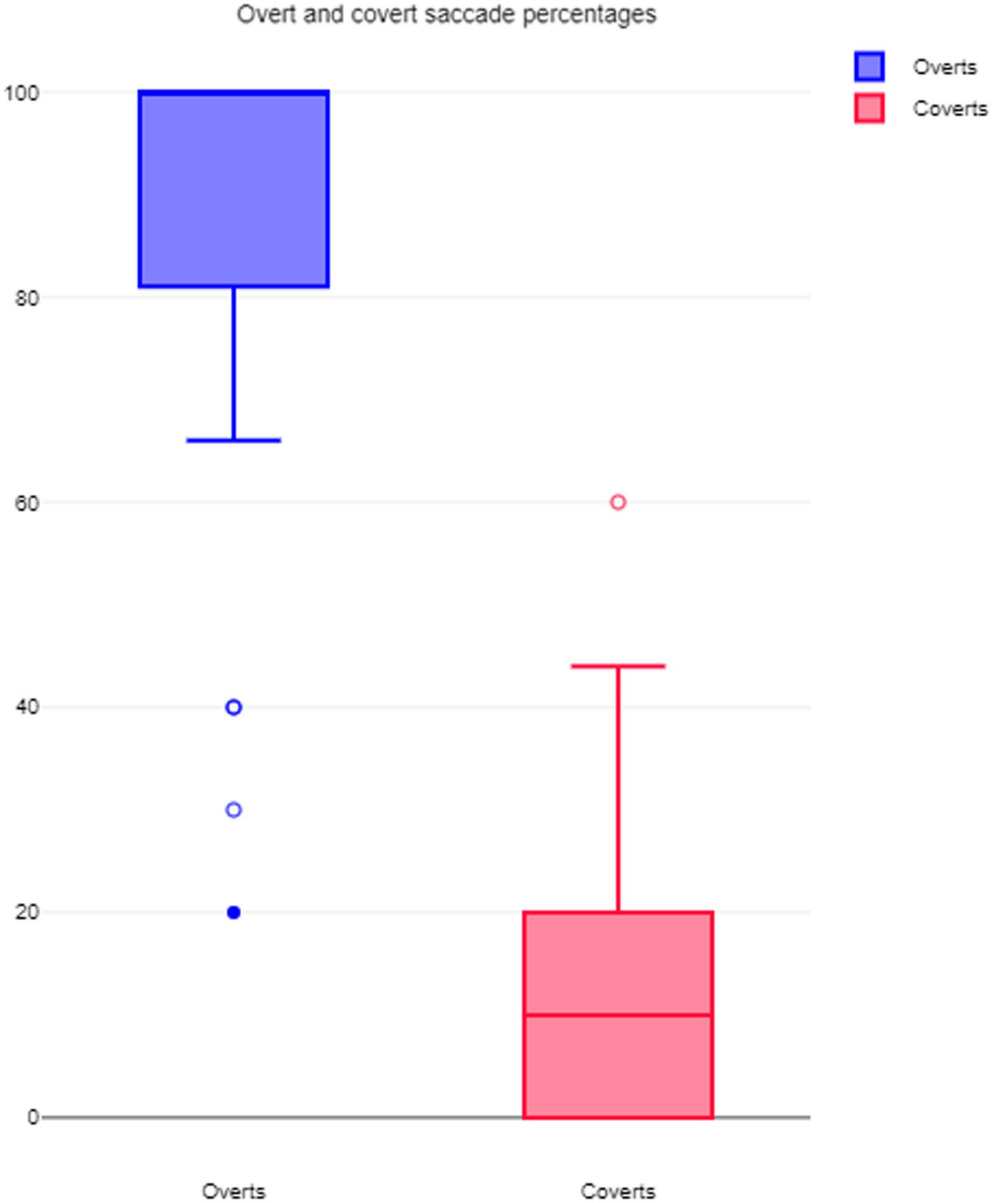
Distribution of overt and covert saccades.

On the other hand, all 88 ears generated overt saccades (100%) in SHIMP with an average percentage of 93% times of such saccades generated during the head thrusts. Figure 4 and Table 5 demonstrate the relative distribution of covert saccades and overt saccades.

### Peak saccadic velocity of overt saccades

**Figure 5:**
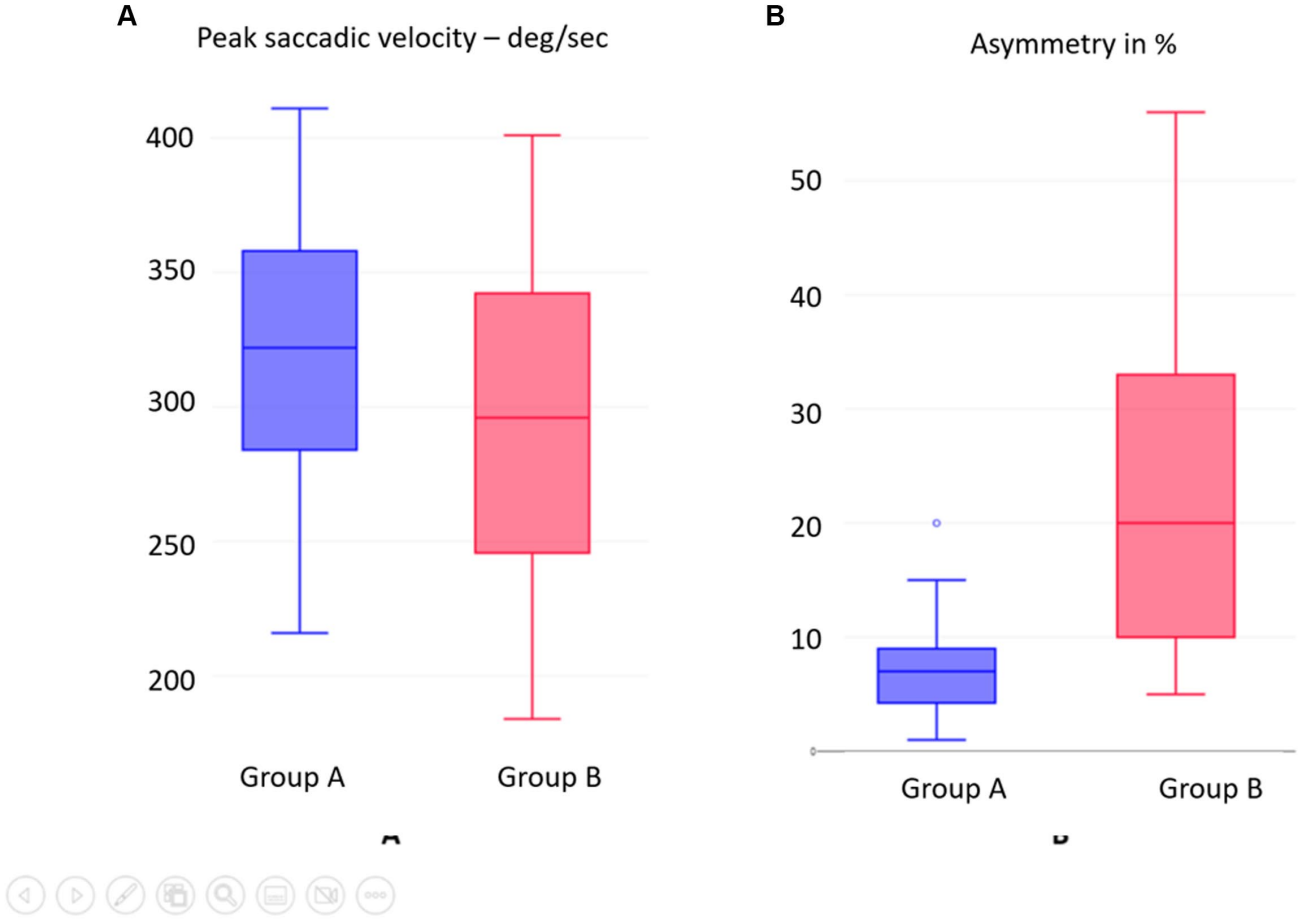
**(A)** SHIMP overt saccades peak saccadic velocity PSV compared between groups. **(B)** SHIMP VOR gain asymmetry between Groups A and B.

In Group A, the normal group, the average peak saccadic velocity of overt saccades measured was 315.39^0^/s +/−56.3, while in Group B, the peak saccadic velocity of overt saccades measured was 293.6^0^/s +/−56.76. Statistically, the values differed from each other in a significant way with a moderate effect size (*p* = 0.03845, *d* = 0.3). Table 5 and Figure 5A show the distribution of the difference. A wide range of PSV was observed in Group B.

### Latencies of SHIMP covert and overt saccades

The latencies of SHIMP covert saccades were 113.29+/−24.66 milliseconds in Group A and 111.63+/−21.26 milliseconds in Group B that did not differ significantly from each other (*p* > 0.05). The latencies of SHIMP overt saccades in Group A were 215.68+/−46.16 milliseconds and 216.74+/−37.54 milliseconds in Group B. Again, this did not achieve statistical significance (*p* > 0.05). Table 5 demonstrates this.

### Asymmetry of SHIMP VOR gain between the sides

The asymmetry of SHIMP VOR gain between the two sides in the normal Group A was 7.42+/−4.68%, while in the abnormal group B, it was 21.12+/−14.42. This was statistically significantly different from each other with a large effect size (*p* = 0.000136; *d* = 1.28). Table 5 shows this, and Figure 5B illustrates the distribution. This asymmetry such as the SHIMP VOR gain and the PSV showed a wide range of distribution.

### Group B1

Seven children in the cohort belonging to Group B were designated as Group B1 who showed normal vHIT VOR gain but also showed covert and overt saccades. All these children were diagnosed with vestibular pathologies with concomitant SNHL in cochleovestibular conditions, vestibular nerve hypoplasia, Chavda type 2 cross arterial compression of the VIII nerve bundle in the CPA, vestibular neuritis, and vestibular migraine. In SHIMP, the average VOR gain in this group summated on both sides was 0.86+/0.15, and their average asymmetry was 13.71+/−8.2%. When compared to the normal group, the difference in VOR gain was statistically significant with *p* = 0.00001 and a large effect size *d* = 0.97. When compared to the normal group, the difference in asymmetry was statistically significant with *p* = 0.01 with a large effect size *d* = 0.94.

### Group B2

This group consisted of three children who all sustained a central pathology—an arteriovenous malformation in the right cerebellopontine angle with hemorrhage and lower six cranial nerve palsies, a possible pontocerebellar hypoplasia in Stickler’s syndrome and a CACNA1 cerebellar ataxia syndrome. We observed that these three children were unable to generate overt anticompensatory saccades during the head thrusts at the same percentage as the normal or even as the other children with abnormal vestibular function. On an average, they could only generate a saccade on 30% of the head thrusts where all other children did so in 93% of the times. All these children were also positive on the chair VOR suppression test with clearly discernible saccadic catch-ups while rotated with their eyes focussed on a target that moved with the chair (their own index fingers touching each other).

### Compatibility with audit standards

Our audit fulfilled all the criteria and standards set out in methods in terms of establishing our own SHIMP laboratory norms in children with normal vestibular function that agreed with existing literature taking into account SHIMP VOR gain, PSV, latency, and asymmetry in addition to a lesser SHIMP VOR gain as compared to HIMP. The abnormal group in our audit differed significantly in VOR gain, PSV, and asymmetry but not latency as compared to the normal group that we included in our audit standards. The test was easy to perform and could be eminently performed in all children in the audit.

### Illustrative cases

#### Child 1 (Group A)

**Figure 6:**
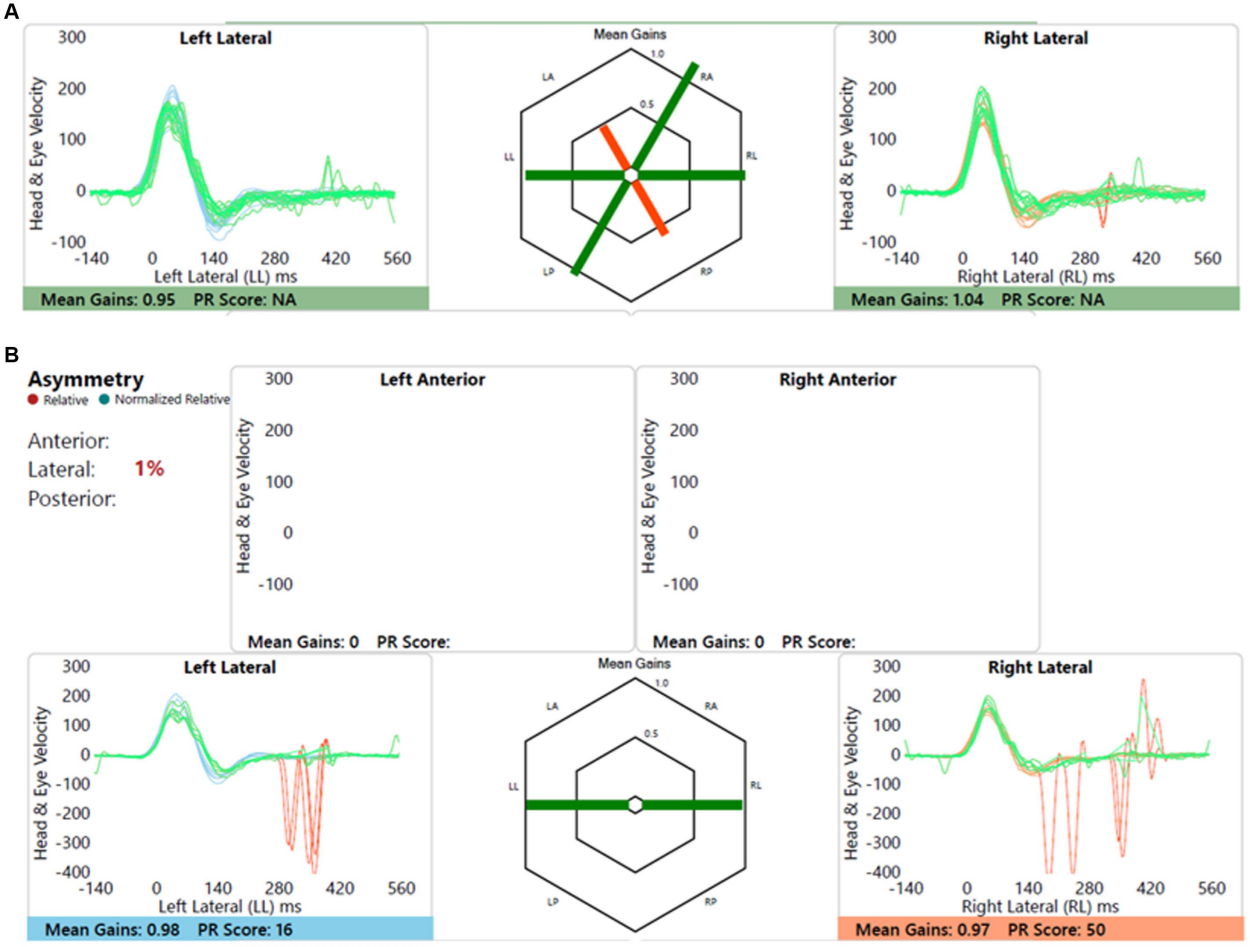
Group A representative – **(A)** HIMP gain = 0.95/1.04 (L/R); **(B)** SHIMP gain = 0.98/0.97 (L/R); PSV = 348/369 ms (L/R); asymmetry: 1%.

Child with bilateral moderate cookie bite sensorineural hearing loss, likely genetic, normal imaging without any vestibular or balance symptoms. This is shown in Figure 6.

#### Child 2 (Group B)

**Figure 7:**
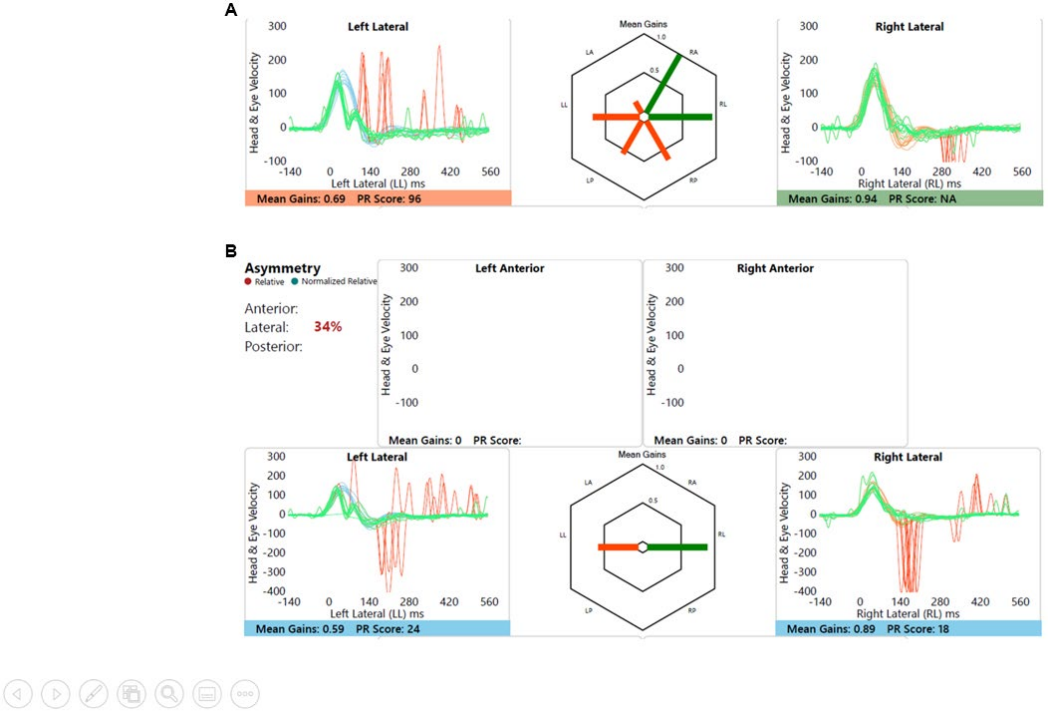
Group B representative – **(A)** HIMP gain = 0.69/0.94 (L/R); **(B)** SHIMP gain = 0.59/0.89 (L/R); PSV = 296/401 ms (L/R); asymmetry: 34%.

Left sided sensorineural hearing loss with delayed motor development and imbalance, and imaging showed left cochleovestibular dysmorphia. This is shown in Figure 7. Note the anticompensatory eye movements (AQEMs) on the healthy side that have now been reported in the contralesional side of vestibular impairment (18), in this case the right. This phenomenon has not been studied in children as yet.

#### Child 3 (Group B1)

**Figure 8:**
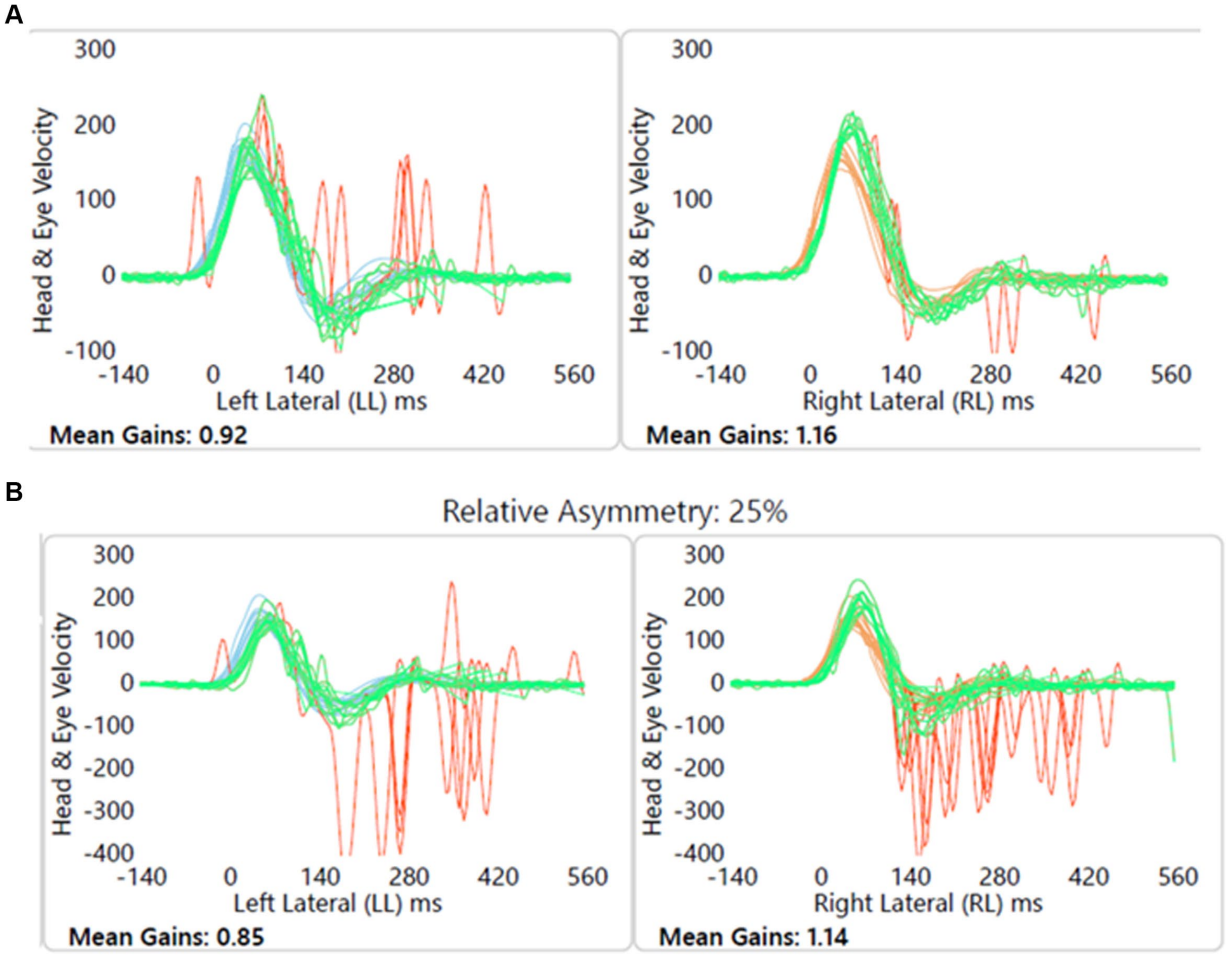
Group B1 representative – **(A)** HIMP gain = 0.92/1.16 (L/R); **(B)** SHIMP gain = 0.85/1.14 (L/R); PSV = 336/245 ms (L/R); asymmetry: 25%; note the normal VOR gain with covert and overt saccades on the left.

Child with no balance or vestibular issues, conductive hearing loss due to otitis media with effusion, left sided craniofacial dysmorphic features, and imaging showed left vestibular nerve hypoplasia. Figure 8 shows this.

#### Child 4 (Group B2)

**Figure 9:**
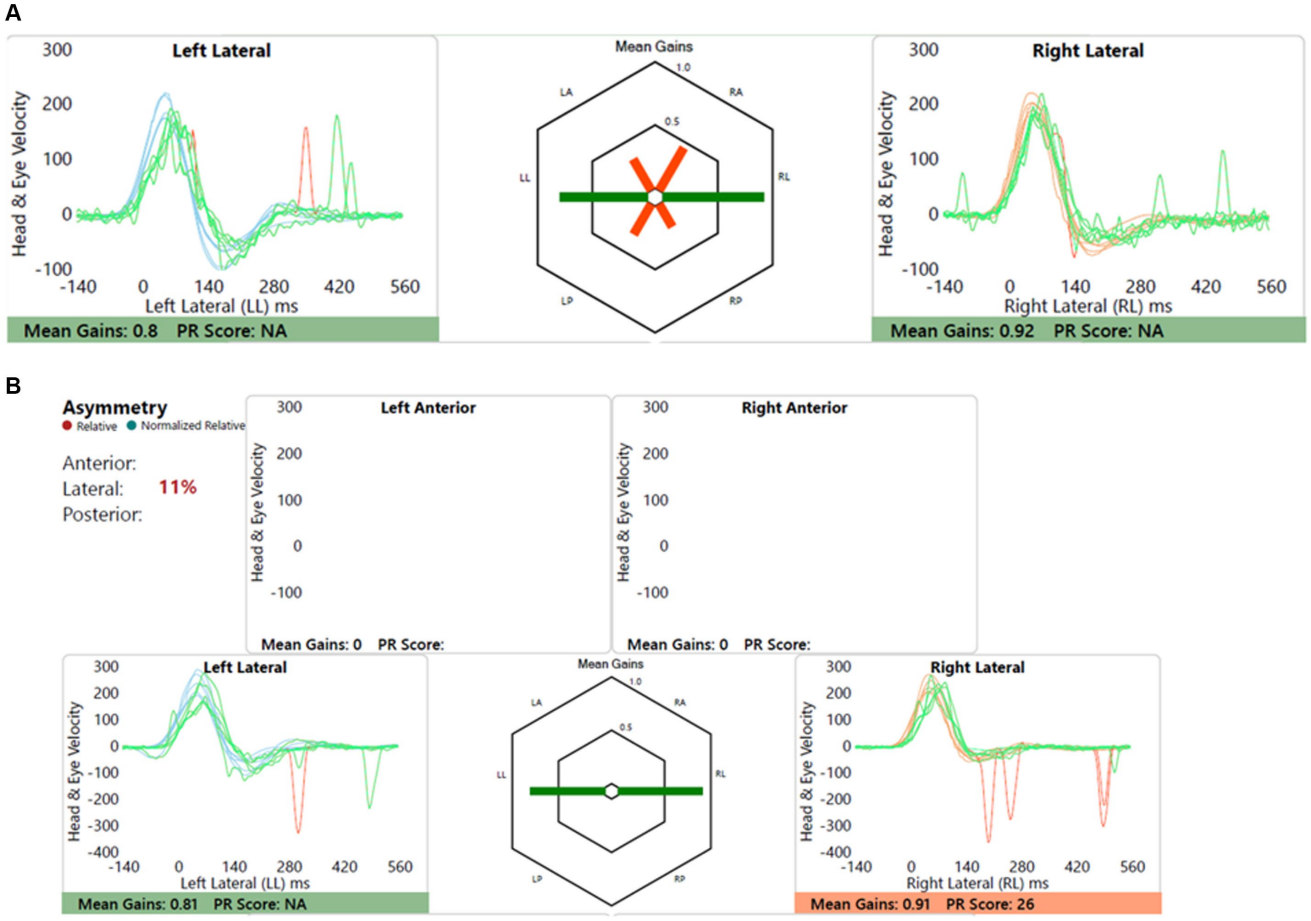
Group B2 representative – **(A)** HIMP gain = 0.61/0.86 (L/R); **(B)** SHIMP gain = 0.67/0.81 (L/R); PSV = 217/201 ms (L/R); asymmetry: 17%; note the lack of overt saccades in SHIMP.

Child with dizziness, ataxia and motor development delay and incoordination, and cerebellar involvement with CACNA1 mutation induced cerebellar ataxia. Figure 9 shows this.

## Discussion

The suppression head impulse test using the SHIMP paradigm and measured by the video head impulse test is a new test to investigate vestibular weakness and compensation. Its utility has been established in published studies (12). In children, SHIMPs have hardly been studied. Only two publications have studied SHIMPs in children and young adolescents in the normal population (15, 16). The present study is the first one of its kind that records SHIMP parameters in a cohort of children with peripheral and central vestibular pathologies.

The minimum head velocity required for either a HIMP or a SHIMP is 120^0^/s to 150^0^/s or above with a small passive head impulse or thrust (19, 20). Therefore, to make any study robust, consistencies in maintaining this head velocity need to be maintained. In children, this may be difficult due to incompatibility with instructions, and thus, it is important to instruct the children correctly. In the present study, the first author, who is an experienced pediatric audiovestibular physician, performed all the SHIMPs with passive head velocities over a narrow range and agreeing to the accepted velocities (154.34^0^/s +/− 20.68).

Evidence about the HIMP paradigm in children is limited but emerging (9). The procedure can be safely and effectively executed in children, but the outcome norms may be different (21) and indeed every individual laboratory should have their own norms (14). Vestibular physiology may be different in adults as compared to children; as in the latter, the system is under a process of development and maturation (9). Similarly, in SHIMPs, extrapolating adult norms are inappropriate and should be avoided. This audit established its own departmental norms by studying a group of children with normal vestibular function.

The test was successfully executed in all children studied. Instructing the cohort on how to do the test played a crucial role to allay apprehension with a combination of play and fun. It emerged that unlike the HIMP, children were able to follow a moving target better than a fixed target that has also been observed in adults (13). Predictability of the impulses has been previously investigated and proposed to contaminate saccades and VOR gain in SHIMP (22, 23). Great care needs to be exercised to eliminate predictability in children that can be mitigated to a certain extent by distractibility between the impulses by an experienced clinician as in this study. Furthermore, the test was not time-consuming, and we believe that successful impulses accepted by the software require experience and skill more so in children than in adults.

The SHIMP VOR gain in the cohort with normal vestibular function with a mean of 0.98 in the present study agrees with the similar values observed in other studies (15, 16). This matches the adult gains measured across four different centers with an average of 0.96 (24). Lateral semicircular VOR gain in HIMP in adults and children is similar as well (21, 25).

When the normal vestibular function group was compared to the abnormal vestibular function group in terms of HIMP and SHIMP VOR gains, there was a significant (*p* < 0.05) and a near significant difference (*p* = 0.05) on the right and the left, respectively. This observation was recorded in several studies that had emerged investigating SHIMP (17, 26–28). The reasons could be multifactorial and uncertain, but proposed theories include a dearth of covert saccades in the SHIMP paradigm and the late onset of VOR suppression at 80–100 milliseconds after the onset of the head impulse (24, 26).

There was a statistically significant difference observed between SHIMP VOR gains in Group A (normal vestibular function) and Group B (abnormal vestibular function). SHIMP gains are reduced in peripheral vestibular disorders where HIMP gain is also diminished in terms of both unilateral and bilateral vestibular deficits (19, 29). In studies in adults as reviewed by Manzari (13), this was a consistent finding in established peripheral vestibular pathologies where the SHIMP VOR gains are also less than the HIMP VOR gains.

In this study, the SHIMP VOR gains in the abnormal group B were spread across a much wider area than the gains in the normal group A. This could be an important caveat, and we believe that this is likely due to the variable central VOR gain compensations in children in different stages of compensation and may be quite useful for planning a rehabilitation programme in the future. VOR suppression is governed by central mechanisms and is an example of visual–vestibular interaction that gradually recovers with compensation in subjects with VOR deficit in peripheral vestibular lesions by virtue of central plasticity (13). Therefore, variable SHIMP gains in children with ongoing compensated vestibular weakness augment the premise that vestibular compensation is a dynamic process yielding a range of SHIMP gains with evolution of vestibular compensation over a period of time. Indeed, vestibular rehabilitation is likely to improve the SHIMP gains that can be used to monitor efficacy and outcome of rehabilitation. SHIMP VOR gain in the pathological cohort is thus an important measurement of the state of vestibular compensation.

The SHIMP paradigm is characterized by a singular dearth of covert saccades and is called a covert saccade killer in subjects with peripheral vestibulopathy (24). There were no covert saccades generated in five out of the seven children who exhibited classical vestibular deficit with low HIMP VOR gain and covert/overt saccades. However, there have been studies that had observed covert saccades in SHIMP. These studies observed that covert saccades in SHIMP are far and few in between, and while subjects consistently generated overt saccades (except in acute vestibulopathy), covert saccades are rare (12, 19, 26, 27). In the current study, a third of the children generated covert saccades in the SHIMP paradigm but they were generated only in 6–10% of the head impulses delivered. In contrast, overt saccades were present in almost all children (except Group B2) in both groups with an average of 93% of the impulses delivered that agree with current observations.

An important outcome measure emerging from SHIMP research is the parameter of peak saccadic velocity or PSV. Modern software allows this calculation. Studies are limited in adults as well investigating this. Park et al. observed a high degree of correlation between SHIMP PSV and HIMP VOR gain (29). Shen et al. in their comprehensive study of PSV in normal, unilateral, and bilateral vestibular deficits showed that PSV was significantly diminished in the pathological group (30). This parameter has recently been postulated to be a rather important measure to assess compensation from vestibular deficits providing a useful insight into the compensation in adults (13). The current study highlights some interesting insights into the PSV in children. There was a statistically significant difference between PSV in the normal and abnormal group. However, again to be noted is that the PSV in the abnormal group is spread over a wider area that may suggest that PSV reflects children in various stages of compensation implying the dynamic nature of a changing PSV with compensation. Research is needed to monitor PSV in vestibulopathies over a period of time to get an idea about compensation and factor into an objective rehabilitation outcome.

This study did not observe any significant difference between the groups in the latencies of overt saccades. Latency studies are small in number in SHIMP. Roh et al. (27) observed a similar finding, i.e., latencies across normal and pathological groups do not differ significantly in latencies. This suggests that regardless of the pathology or a normal system, the central VOR suppression mechanism will attempt to maintain the time of generation of the saccade to maximize vestibulo-visual interaction to maintain stable gaze on the retina while tracking a moving target. This study also agreed with latencies observed in the only two studies performed in the pediatric population.

One important advantage of the vHIT paradigms (HIMP and SHIMP) is that it allows assessing one side at a time such as the caloric test. Human physiology shows variations in function between the sides, and it is expected that SHIMP parameters will show variations or asymmetry between the left and the right side. Generally, this is calculated as a percentage with the formula x(R) – x(L)/ x(R) + x(L) x 100 where x represents the VOR gain. Present day software automatically performs this, and in SHIMP, the VOR gain is considered. The average asymmetry in the normal group in our study was 7.42, while in the abnormal group, it was 21.2. The two groups significantly differed from each other. Of course, in symmetrical bilateral vestibular hypofunction, there will be little asymmetry, but no children in the current series showed symmetrical bilateral vestibular weakness. Since asymmetry is due to asymmetrical VOR gains that is lower on the affected side, our result is somewhat expected. However, our study establishes some norms in our laboratory, and it is a rational thought that gain asymmetry like PSV may be a dynamic process indicating different stages in compensation. Again, there is a possibility that this parameter can be incorporated into an objective rehabilitation outcome and requires further research.

There were seven children in Group B designated as Group B1 who had normal HIMP VOR gains but showed saccades. Evidence is emerging that saccades are probably better indicators of past vestibular weakness that is compensated to a certain extent^6.^ All these seven children had diagnosed vestibular pathologies. SHIMP VOR gain was significantly different when compared to the normal group as would be expected regardless of whether SHIMP VOR gain had recovered or not as discussed earlier. However, the observed asymmetry was 13.71 in this group that was significantly different from that in the normal group. This is a rather interesting observation as it suggests that SHIMP VOR gain recovery in unilateral vestibulopathy occurs but might not reach the same level as normal even with compensation. Therefore, in the presence of normal VOR gain and saccades in HIMP, this augments the original premise that there would have been a previous vestibular damage. For children, this is crucial to glean as it leads to situational counseling as to how not to provoke the vestibular system when the system will desaturate leading to symptoms that can be distressing.

There were three children in Group B designated as Group B2 who did not generate as many overt anticompensatory saccades as the other 41 children in the cohort. They all were shown to possess cerebellar lesions on imaging and genetic testing. In fact, the average percentage of overt saccades was only 30% compared to 93% in others. All these three children also generated catch-up saccades on the chair VOR cancellation test. We believe that since the cerebellum plays such a crucial role in integrating VOR suppression (31), a problem in the cerebellum might lead to a fundamental deficit in generating those saccades in the first place. This finding is too early to generalize, but it might be possible in the future to utilize the SHIMP paradigm as an indicator of a cerebellar pathology. Halmagyi et al. in their review of the HIMP (8) had indeed alluded to the use of the vHIT in central lesions. So far, however, the vHIT concentrates mainly on the peripheral vestibular system.

Our study fulfilled all the audit standards that were enumerated at the beginning of the exercise. We observed that the SHIMP paradigm in the vHIT test is user-friendly in children and they tolerate it rather well. Our SHIMP VOR gains and latencies of anticompensatory saccades in children with intact vestibular function follow what has been published elsewhere. In addition, we have highlighted the role of the PSV, asymmetry, and VOR gain in a group of children with abnormal vestibular system. Vestibular testing in children is an art that demands experience and a high skill set. It is essential to glean as much information as possible. The audit led to the recommendation of using SHIMP in all children (over 4 years of age) referred for vestibular assessment in the hospital where the study was conducted. As a result, SHIMP has now been adopted as an indispensable tool in our laboratory to yield meaningful results influencing management of balance problems in children that was the main objective of the audit.

There are several limitations to this study. First, this was a retrospective case note audit to assess logistics, feasibility, and utility of a test to improve services and as such was not randomized and did not carry any hypotheses. Second, the number of children in each group was small, and there were no set inclusion or exclusion criteria as we wanted to audit SHIMPs in all populations where the test was indicated. Third, we did not audit saccade morphology and clustering/dispersion or PR score as there are no norms in the pediatric population. Fourth, the findings of this study cannot be generalized as yet as this was just a snapshot of SHIMP in children. Finally, complex statistical methods, for example linear regression algorithms, correlation studies, sensitivity and specificity assessments, were not performed, rather we resorted to simple statistical methods for description and comparison. Yet, this study provides benchmarks for future audits and established our own laboratory SHIMP norms in children in addition to providing valuable insight to this test in children. We recommend that SHIMP is used in all cases of pediatric vestibular assessment even where the HIMP cannot be relied upon and is used to supplement HIMP. It is hoped that formal research will be instituted investigating SHIMP in children.

## Conclusion

The SHIMP paradigm in the vHIT protocol is eminently feasible in children with good compatibility. Parameters from the test that include VOR gain, PSV, and asymmetry in VOR gains between the sides in compensating vestibular deficits are important measures of vestibular function. The SHIMP supplements the HIMP paradigm, where some outcome measures such as PSV and asymmetry yield added information even in cases where the HIMP VOR gain may be normal. The absence of SHIMP overt saccades may indicate a central cerebellar integration pathway deficit. SHIMP supplements the HIMP and should be used in all children presenting with balance issues.

## Data availability statement

The original contributions presented in the study are included in the article/supplementary material, further inquiries can be directed to the corresponding author.

## Ethics statement

Ethical review and approval was not required for the study on human participants in accordance with the local legislation and institutional requirements. The study was an audit that does not require ethical approval. Written informed consent from the patients/participants or patients/participants’ legal guardian/next of kin was not required to participate in this study in accordance with the national legislation and the institutional requirements.

## Author contributions

SD: Conceptualization, Data curation, Formal analysis, Funding acquisition, Investigation, Methodology, Project administration, Resources, Software, Supervision, Validation, Visualization, Writing – original draft, Writing – review & editing. RC: Conceptualization, Data curation, Formal analysis, Investigation, Methodology, Project administration, Resources, Validation, Writing – review & editing. JW: Formal analysis, Investigation, Methodology, Resources, Validation, Writing – review & editing. AM: Formal analysis, Investigation, Methodology, Resources, Validation, Writing – review & editing. SR: Conceptualization, Data curation, Formal analysis, Investigation, Methodology, Project administration, Resources, Validation, Visualization, Writing – review & editing. LM: Conceptualization, Formal analysis, Methodology, Supervision, Validation, Visualization, Writing – review & editing.

## Footnotes

^1^ https://www.socscistatistics.com/tests/
